# Update on New Aspects of the Renin-Angiotensin System in Hepatic Fibrosis and Portal Hypertension: Implications for Novel Therapeutic Options

**DOI:** 10.3390/jcm10040702

**Published:** 2021-02-11

**Authors:** Indu G. Rajapaksha, Lakmie S. Gunarathne, Peter W. Angus, Chandana B. Herath

**Affiliations:** 1 Department of Medicine, The University of Melbourne, Austin Health, Heidelberg, VIC 3084, Australia; indu.rajapaksha@unimelb.edu.au (I.G.R.); lakmie.gunarathne@unimelb.edu.au (L.S.G.); 2 Department of Gastroenterology and Hepatology, Austin Health, Melbourne, VIC 3084, Australia; peter.ANGUS@austin.org.au; 3 South Western Sydney Clinical School, Faculty of Medicine, Ingham Institute for Applied Medical Research, University of New South Wales, Liverpool, NSW 2170, Australia

**Keywords:** liver fibrosis and cirrhosis, portal hypertension, renin angiotensin system, angiotensin converting enzyme 2, angiotensin-(1–7), Mas related G protein-coupled receptor type-D

## Abstract

There is considerable experimental evidence that the renin angiotensin system (RAS) plays a central role in both hepatic fibrogenesis and portal hypertension. Angiotensin converting enzyme (ACE), a key enzyme of the classical RAS, converts angiotensin I (Ang I) to angiotensin II (Ang II), which acts via the Ang II type 1 receptor (AT1R) to stimulate hepatic fibrosis and increase intrahepatic vascular tone and portal pressure. Inhibitors of the classical RAS, drugs which are widely used in clinical practice in patients with hypertension, have been shown to inhibit liver fibrosis in animal models but their efficacy in human liver disease is yet to be tested in adequately powered clinical trials. Small trials in cirrhotic patients have demonstrated that these drugs may lower portal pressure but produce off-target complications such as systemic hypotension and renal failure. More recently, the alternate RAS, comprising its key enzyme, ACE2, the effector peptide angiotensin-(1–7) (Ang-(1–7)) which mediates its effects via the putative receptor Mas (MasR), has also been implicated in the pathogenesis of liver fibrosis and portal hypertension. This system is activated in both preclinical animal models and human chronic liver disease and it is now well established that the alternate RAS counter-regulates many of the deleterious effects of the ACE-dependent classical RAS. Work from our laboratory has demonstrated that liver-specific ACE2 overexpression reduces hepatic fibrosis and liver perfusion pressure without producing off-target effects. In addition, recent studies suggest that the blockers of the receptors of alternate RAS, such as the MasR and Mas related G protein-coupled receptor type-D (MrgD), increase splanchnic vascular resistance in cirrhotic animals, and thus drugs targeting the alternate RAS may be useful in the treatment of portal hypertension. This review outlines the role of the RAS in liver fibrosis and portal hypertension with a special emphasis on the possible new therapeutic approaches targeting the ACE2-driven alternate RAS.

## 1. Introduction

Fibrosis or scarring of the liver is initiated as a part of the wound healing response to tissue injury. The end result of chronic fibrotic injury to the liver is cirrhosis, in which there is extensive scar formation, distortion of liver parenchyma by septae and nodule formation, and alterations in blood flow and this can finally lead to liver failure [1,2]. A major outcome of cirrhosis is the development of portal hypertension which is responsible for many of the complications including life-threatening variceal bleeding [3]. Cirrhosis has become the 11th most common cause for deaths in humans and was responsible for approximately 1.2 million deaths worldwide in 2016 [4]. The most common causes for chronic liver disease (CLD) include chronic viral infections (e.g., hepatitis B and C), excessive alcohol consumption, non-alcoholic fatty liver disease (NAFLD) and cholestatic diseases such as primary biliary cholangitis and primary sclerosing cholangitis [2,5,6]. 

Despite being a major health problem, there is no specific medical treatment for cirrhosis. Therefore, treatments that target the causative factors and managing complications associated with cirrhosis including portal hypertension are the only currently available options. Thus, if the causative agent is viral hepatitis C (Hep C), treatment with antiviral therapies leads to cessation and even reversal of tissue fibrosis [7]. Treatment options for established cirrhosis and portal hypertension are limited with the major therapy being non-selective beta-blockers (NSBB). Recent research shows that the renin angiotensin system (RAS) is activated during the development of cirrhosis and contributes to the pathogenesis of both liver fibrosis and portal hypertension [3,6]. This review presents an update on new aspects of the RAS, with special emphasis on novel mechanisms via which the RAS can be manipulated. 

### 1.1. The Renin Angiotensin System (RAS) 

In normal physiology, the RAS plays a very important role in vascular resistance and regulation of blood pressure, sodium and water homeostasis, and tissue remodeling during a tissue injury. The RAS comprises two arms known as the “classical arm” and the “alternate arm” (protective arm). The alternate arm of the RAS plays a major role in counter-balancing many of the deleterious effects of the classical RAS [8]. 

#### 1.1.1. The Classical Arm of the Renin Angiotensin System 

The classical arm of the RAS can be thought of as a linear cascade where angiotensinogen is converted to the effector peptide of the system, angiotensin II (Ang II). Angiotensinogen is produced mainly in the liver by hepatocytes and released into the circulation. Circulating angiotensinogen is converted to the decapeptide angiotensin I (Ang I) by renin, an enzyme produced by the juxtaglomerular apparatus of the kidney. Thereafter, angiotensin converting enzyme (ACE), mainly present in the lungs, converts Ang I to Ang II [9]. There is also an ACE independent pathway of Ang II production from Ang I, which is regulated via a serine endopeptidases named chymase [10,11]. Ang II mediates its effects through two G-protein-coupled receptors, Ang II type 1 receptor (AT1R) and Ang II type 2 receptor (AT2R). AT1R is the predominant receptor type during adult life whereas AT2R, although having some functional role in adults, has been postulated to function mainly during fetal life [12]. 

Ang II by binding to the AT1R mediates classical RAS functions which include vasoconstriction (by directly affecting vascular smooth muscle cells), sodium homeostasis (by increasing sodium reabsorption through renal tubules and by stimulating the adrenal gland to release aldosterone), increasing thirst (by acting on AT1R in the brain), induction of inflammation and the wound healing response via secretion of cytokines, chemokines, and extracellular matrix proteins. Ang II also acts as a prooxidant and prothrombotic agent and interferes at several steps of intracellular insulin signaling pathways such as the PI3 kinase (phosphoinositide 3-kinase) and MAP (mitogen activated protein kinase) kinase pathways [13,14]. 

#### 1.1.2. The Alternate Arm of the Renin Angiotensin System

Whilst the physiological role of the classical RAS is well-established, the discovery of a new RAS enzyme, ACE2, a homologue of ACE, has dramatically changed our understanding of the RAS physiology [15,16]. The ‘alternate or the protective arm’ of the RAS driven by ACE2 is considered as the counter-regulatory arm of the classical RAS [3]. 

**Figure 1:**
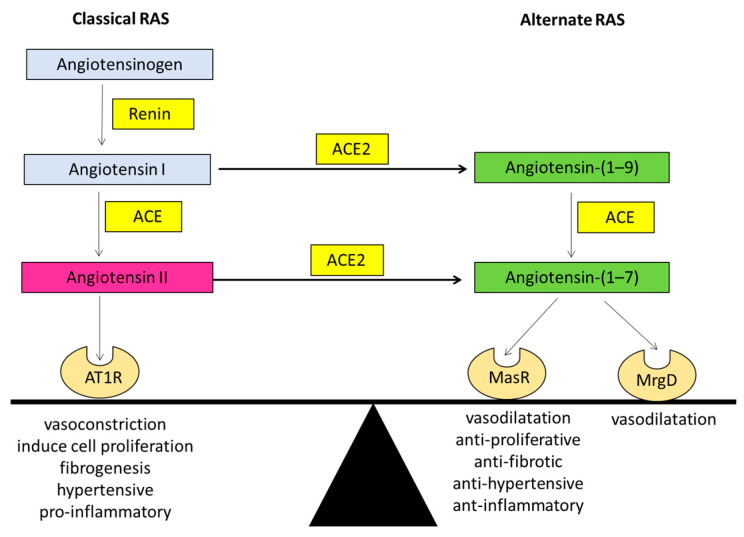
The ‘balance’ between the two arms of the renin angiotensin system (RAS). Graphical representation of the classical arm (ACE/Angiotensin II/AT1R) and the alternate arm (ACE2/Angiotensin-(1–7)/MasR) of the RAS where the alternate arm counter-balances the deleterious effects of the classical arm. Angiotensin II can exert its effects via the angiotensin II type 1 receptor (AT1R). Whilst angiotensin-(1–7) of the alternate RAS acts mainly via the Mas receptor (MasR), recent evidence suggests that it also transduces its signal via the Mas related G protein-coupled receptor type-D (MrgD). ACE: angiotensin converting enzyme; ACE2: angiotensin converting enzyme 2.

In the late 80s, researchers discovered a biologically active heptapeptide angiotensin-(1–7) (Ang-(1–7)) of the RAS [17]. The enzymatic pathway responsible for producing Ang-(1–7) came to light when ACE2 was discovered in the year 2000 by two independent laboratories [15,16]. It is now known that Ang-(1–7) peptide is produced by ACE2 after cleavage of a carboxyl terminal single amino acid from the 8-amino acid peptide Ang II. ACE2 is a zinc-metalloproteinase and a type-1 transmembrane protein which consists of 805 amino acids with a single transmembrane alpha-helical portion, an extracellular N-terminus portion containing the catalytically active domain and an internal inactive C-terminus [9]. ACE2 is structurally similar to ACE; however, it is functionally different with different substrate affinities than that of ACE, and ACE2 is resistant to ACE inhibitors (ACEi). The major action of ACE2 is to break down Ang II to Ang-(1–7), which acts through the Mas receptor (MasR), a G protein-coupled receptor (GPCR). Ang (1–7) is subsequently metabolized via ACE into Ang-(1–5) and via the other neutral endopeptidases (NEP), neprilysin into Ang-(1–4) [18]. The ACE2/Ang-(1–7)/MasR arm counter-regulates many of the actions of the classical RAS, thus producing opposing effects to those of the classical RAS, including antihypertensive, anti-inflammatory, antithrombotic, antiproliferative, and antifibrotic effects (Figure 1). 

Although the MasR is recognized to be the functional receptor for Ang-(1–7) [19], Santos and colleagues in 2003 suggested that certain effects of Ang-(1–7) may not be mediated through MasR [20]. This was supported by the finding that MasR blocker D-Ala^7^-Ang-(1–7) (A779) could not block the Ang-(1–7) mediated vasodilatation in the rat aorta, however, these effects were completely blocked by the Ang-(1–7) antagonist D-Pro^7^-Ang-(1–7) (D-Pro) [21]. Subsequently, a study by Gembardt and colleagues showed that upon stimulation with Ang-(1–7), COS cells overexpressing MasR or a newly characterized Ang-(1–7) receptor, the Mas related G protein-coupled receptor type-D (MrgD), released arachidonic acid [22]. Lautner and colleagues later showed that in addition to Ang-(1–7), the RAS peptide alamandine also activates the MrgD, and the vasodilatory effects produced by alamandine were blocked by the MrgD blocker D-Pro [23]. Work from our laboratory provided further evidence by showing that D-Pro blocked Ang-(1–7) mediated vasodilatation in perfused cirrhotic rat livers whilst the MasR blocker A779 had no effect [24].

In a recent study, it was shown that when transfected with either the MasR or MrgD, mesangial cells release cyclic adenosine monophosphate (cAMP) in the presence of Ang-(1–7) [25]. Moreover, in MasR or MrgD transfected HEK293 cells, blockade of MasR and MrgD with A779 and D-Pro, respectively, abolished the release of cAMP. Moreover, the effects of MrgD stimulation in-vitro were confirmed by in-vivo functional studies by showing that the hemodynamic responses to a bolus injection of Ang-(1–7) were blunted in MrgD knockout (MrgD-KO) mice compared with the controls. With these findings, it is now considered that the alternate arm of the RAS consists of ACE2/Ang-(1–7)/MasR/MrgD [25]. 

### 1.2. The Role of Classical RAS in Liver Fibrosis

Traditionally, the RAS was considered as an endocrine system. However, recent studies have shown that there is a local RAS which functions in an autocrine and/or paracrine manner, in major organs such as heart, the kidneys and liver [26,27]. The local RAS is activated in response to a tissue injury, which leads to a wound healing response with upregulated expression of the RAS components at the site of repair [28]. Initially it was discovered that the classical RAS plays an important role in tissue repair and organ fibrosis in heart disease and chronic kidney disease (CKD), and RAS inhibitors have been shown to have benefits in these conditions beyond those that are due to their antihypertensive effects [29,30]. 

There is also substantial evidence that Ang II is a major mediator in hepatic fibrosis. Serum Ang II concentration is significantly elevated in patients with cirrhosis [31,32] and the local RAS in the liver is activated as a response to the chronic injury. Findings from our laboratory and others showed that the hepatic expression of the classical RAS comprising ACE, Ang II and AT1R is increased in the diseased liver compared to those in healthy livers [3,33,34,35,36,37]. Moreover, these studies reported that the expression of the RAS components was localized to the areas of active fibrogenesis, suggesting that the local classic RAS plays a major role during the progression of liver fibrosis.

Following liver injury a major cell type responsible for the wound healing response is the hepatic stellate cell (HSC) [38]. Ang II plays a major role in the activation and phenotypic transformation of HSCs into active myofibroblasts which drive tissue fibrosis. Whilst quiescent HSCs have minimal expression of the RAS components and do not produce Ang II, activated HSCs express all components of RAS including angiotensinogen, renin, ACE, and AT1R [28]. Activated HSCs thus have the potential to synthesize Ang II, which acts on AT1R in an autocrine fashion [28,39,40], stimulating their proliferation [41]. The molecular mechanism responsible for HSC activation appears to be the activation of nicotinamide adenine dinucleotide phosphate (NADPH) oxidase, an enzyme which produces reactive oxygen species (ROS) in response to Ang II. Inhibition of NADPH oxidase with diphenylene iodonium (DPI) blocked Ang II-induced ROS production in cultured HSCs, confirming that NADPH oxidase mediates the increase in ROS after Ang II stimulation [31]. This evidence suggests that Ang II has the potential to induce HSCs to produce ROS that can further stimulate the fibrogenic process in an autocrine and/or paracrine manner. In addition, Ang II acts as a powerful chemoattractant for activated HSCs [31]. Thus, Ang II becomes a contributing factor for HSC proliferation, activation and accumulation at the site of injury. 

Furthermore, Ang II exerts a direct influence on endothelial function [13]. In general, normal endothelial function is determined by the cell redox state, which is controlled by a homeostatic balance between nitric oxide and ROS. Griendling and colleagues in 1994 showed that prolonged exposure of vascular smooth muscle cells (VSMCs) to Ang II induced oxidative stress and ROS generation due to activation of NADPH, the enzyme responsible for intracellular superoxide generation. In addition, it has been shown that Ang II stimulates the release of endothelin-1, a potent vasoconstrictor and inducer of smooth muscle cell proliferation in vasculature, while altering the balance between the fibrinolysis and coagulation processes [13]. 

**Figure 2:**
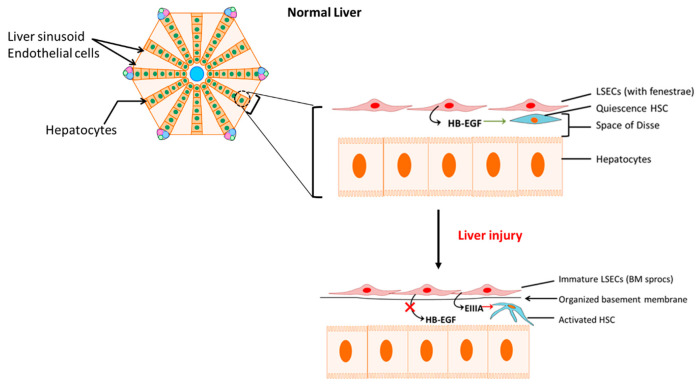
Activation of hepatic stellate cells (HSCs) by immature liver sinusoidal endothelial cells (LSECs). Reduced release of heparin-binding epidermal growth factor (HB-EGF) with concomitant increase in fibronectin isoform EIIIA release by bone marrow-derived immature LSECs activates HSCs. BM sprocs, bone marrow (BM) derived sinusoidal endothelial cell progenitor cells (sprocs). BM: bone marrow; HB-EGF: heparin-binding epidermal growth factor.

When normal liver sinusoidal endothelial function is disturbed by factors such as Ang II induced ROS generation, the damaged liver sinusoidal endothelial cells (LSEC) are replaced by bone marrow (BM) derived sinusoidal endothelial cell progenitor cells (sprocs) in order to compensate for the loss. These BM sprocs which lack LSEC fenestrae arrange along the sinusoids and an organized basement membrane is laid down. This process of dedifferentiation of injured LSECs leads to “capillarization” of the sinusoids [42]. Although normal LSECs are responsible for the production of heparin-binding epidermal growth factor (HB-EGF), which maintains HSC quiescence (Figure 2), the immature LSECs derived from BM sprocs have less or reduced capacity to release HB-EGF which leads to activation of HSCs [42,43]. In addition, dedifferentiated LSECs express EIIIA fibronectin isoform which influences quiescence HSCs to change their phenotype to the activated form [44,45]. Thus, LSECs perform a critical crosstalk function in the HSC activation process in hepatic fibrosis by simultaneous reduction in HB-EGF and expression of EIIIA (Figure 2).

#### ACE Inhibitors (ACEi) and Angiotensin II Type 1 Receptor Blockers (ARBs) in Liver Fibrosis

Since Ang II plays a major role in liver fibrosis, ACEi and ARBs have been studied as potential antifibrotic therapies in liver disease [40]. ACE inhibitors are widely used in patients with high blood pressure and chronic heart failure. Studies in preclinical models suggest that ACEi attenuate the expression of transforming growth factor beta 1 (TGF-β1), collagen and other extra cellular matrix (ECM) proteins including matrix metalloproteinase (MMP)-2 and MMP-9, leading to attenuation of fibrosis [13,46,47]. 

There have also been several animal studies which have examined the effects of ARBs in liver diseases. Studies in bile duct ligated (BDL) rats have shown that ARB, telmisartan decreased gene expression of ACE, AT1R, collagen type III, and TGF-β1 while increasing the expression of ACE2 and MasR with concomitant reduction in hepatic fibrosis [48,49]. Another ARB, losartan, significantly ameliorated the progression of hepatic fibrosis induced by carbon tetrachloride (CCl_4_) in rats along with significant reductions in gene expression levels of AT1R and TGF-β1 [50]. Moreover, a study performed in rats with NASH induced by feeding methionine-choline deficient (MCD) diet showed that another ARB, olmesartan, attenuated serum aspartate transaminase (AST) levels, HSC activity, oxidative stress, gene expression of TGF-β1 and collagen, collectively leading to improved liver fibrosis likely resulting from reduced activation of HSCs [51]. 

Despite evidence from animal studies there is a relative lack of clinical studies of RAS inhibition in human liver fibrosis. A retrospective study carried out by Corey and colleagues suggested a reduction in liver fibrosis and necroinflammation in patients with chronic hepatitis C who received ACE inhibitors and ARBs as a treatment for hypertension [52]. The study reviewed 284 liver biopsies from the patients who were evaluated at an outpatient hepatology clinic during a 5 year period. The patients were classified into three groups: Group 1—hypertensive patients (*n* = 143) who received ACE inhibitors (captopril, enalapril, lisinopril, quinapril, and trandolapril) or ARBs (losartan, valsartan and irbesartan); Group 2—hypertensive patients (*n* = 91) treated with β-adrenergic antagonists, calcium channel antagonists, diuretics, α-adrenergic antagonists, and vasodilators; Group 3—patients (*n* = 50) with chronic hepatitis C infection who did not have hypertension. The results showed a significantly low mean Ishak fibrosis score in patients included in the Group 1 who received ACE inhibitors and ARBs compared to the patients in Group 2 who were treated with other treatments for hypertension. The patients with no hypertension included in the Group 3 showed the lowest mean fibrosis score supporting the theory that hypertension and activation of RAS contributes to fibrosis progression. Another register-based cohort study of patients (*n* = 70,546) with a first-time diagnosis of chronic liver disease between 2005 and 2012 in Sweden revealed a marked reduction in liver-related mortality among patients with alcoholic liver disease who received ACE inhibitors [53]. 

Only a small number of randomized studies have evaluated the effects of RAS blockade in liver fibrosis. A randomized open-label controlled study performed by a group of researchers from South Korea in 2012 investigated the antifibrotic effect of ARBs in patients with compensated alcoholic liver disease [54]. They treated 42 patients with candesartan and ursodeoxycholic acid (UDCA), and the control group (*n* = 43) received UDCA alone for 6 months. There was a reduction in Laennec fibrosis score, area of fibrosis, hydroxyproline level, and α-smooth muscle actin in the candesartan treated group. Another randomized clinical trial performed in 2011 in a group of selected patients with cirrhosis compared those who received (*n* = 24) and those who did not receive (*n* = 24) olmesartan treatment for one year. There was a reduction in TGF-β1 in the olmesartan treated group but not in hepatic fibrosis markers which include serum hyaluronic acid, type IV collagen, and procollagen III N-terminal propeptide levels [55]. In 2007, Debernardi-Venon and colleagues from Italy published data from a randomized clinical trial of candesartan treatment conducted in 47 compensated Child A and Child B cirrhotic patients. The results showed a significant reduction in serum fibrosis marker hyaluronic acid in candesartan treated patients compared to the untreated patients [56]. Although there is a reduction in hyaluronic acid level in treated patients, the results were not supported by histological data. Between 2004 and 2006, a 48-month follow up study was conducted on 89 patients with cirrhosis associated with hepatocellular carcinoma (HCC) in Japan using combination therapy with branched-chain amino acid (BCAA) granules and an ACE inhibitor, perindopril [57]. The serum fibrosis markers hyaluronic acid and type IV collagen 7S were measured in groups of patients who received a combination therapy with BCAA granules and perindopril and two single-treatment groups who received either perindopril or BCAA and compared to a control group. The patients included into this study were confirmed to be free of any residual HCC, alcohol consumption and also the status of insulin resistance (IR). The results of this study show that combination therapy of BCAA and perindopril improved serum fibrosis markers as compared to either ACE inhibitor or BCAA alone. Another randomized study conducted in 30 patients with early stages of chronic hepatitis C in Japan showed that oral losartan and UDCA administration has the potential to reduce serum type IV collagen and TGF-β1 compared to UDCA alone, but not liver fibrosis as indicated by METAVIR fibrosis score [58]. A recent double-blind randomized-controlled trial which was designed to study the efficacy of losartan to reduce or reverse the progression of fibrosis in patients with nonalcoholic steatohepatitis (NASH) was unable to show a positive outcome due to the widespread use of ACE inhibitors and ARBs in patients with NASH [59].

Thus, there are some studies which suggest beneficial effects of ACE inhibitors and ARBs as antifibrotic agents, [52,53,57]; however, the evidence is conflicting and there is a definite need for further large randomized placebo controlled clinical trials [60]. In addition, it should be noted that there are also concerns about the use of RAS inhibitors in advanced cirrhosis [61,62] as they can induce arterial hypotension and renal function impairment. 

### 1.3. Role of the Alternate RAS in Hepatic Fibrogenesis

**Figure 3:**
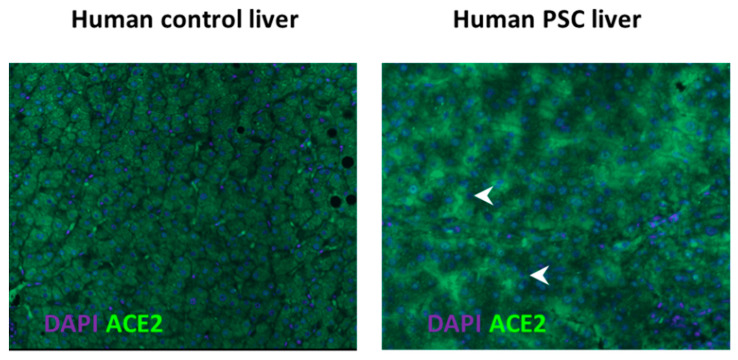
ACE2 protein expression in the liver of healthy controls and in patients with primary sclerosing cholangitis (PSC). Angiotensin converting enzyme (ACE2), the expression of which is very low in healthy livers (left panel), is upregulated in the cirrhotic livers of patients with primary sclerosing cholangitis (PSC) (right panel). Upregulated ACE2 may be important in counter-regulating the ACE and angiotensin II-driven profibrotic effects of the classical renin angiotensin system. Arrowhead, ACE2 staining; DAPI, nuclear staining; magnification, ×100.

Our laboratory has demonstrated that along with the classical arm, the alternate arm is also activated in liver injury [33,35]. The activated alternate arm would be expected to counter the harmful effects produced by the activated classic arm (Figure 1). It has been shown that the components of the classical RAS including angiotensinogen, ACE, and AT1R are upregulated in the areas of active fibrogenesis within one week of bile duct ligation in experimental cholestasis using BDL rats. The expression of alternate RAS components such as ACE2 and MasR were observed at the third week post-BDL whereas, once activated, their expression parallels the changes in the expression of classical RAS components [35]. This was followed by high levels of Ang-(1–7) in the circulation [35]. In line with these findings, ACE2 was found to be increased in patients with liver disease (Paizis et al., 2005). In agreement with these findings the patients with CLD show increased levels of circulating Ang-(1–7) and ACE2 [32,63]. Figure 3 shows high level of ACE2 protein expression in liver specimen collected from patients with primary sclerosing cholangitis (PSC) in comparison with healthy human livers [64]. 

Inhibition of the components of classical arm of the RAS has been extensively tested in animal models of diseases; however, only few studies have investigated the role of the alternate arm of the RAS in liver fibrosis. Emerging evidence suggests that the alternate arm of the RAS is a potential target for research and subsequent drug intervention. One possible way of achieving a therapeutic outcome in liver fibrosis would be to increase the level of antifibrotic peptide Ang-(1–7), which opposes most of the deleterious effects of Ang II. Studies using BDL rats and cultured rat HSCs have confirmed that Ang-(1–7) peptide reduce matrix formation which in turn leads to pronounced improvement in hepatic fibrosis [32]. Moreover, the same study showed that the nonpeptide MasR agonist, AVE0991, significantly decreased the α-SMA protein content and collagen production in rat HSCs. These effects were inhibited by pretreatment with the MasR antagonist D-Ala^7^-Ang-(1–7) (A779), suggesting that the antifibrotic effects of Ang-(1–7) are mediated via the MasR [32]. However, Ang-(1–7) is a peptide that cannot be administered orally. To circumvent this problem, Ang-(1–7) oral formulation has been recently developed with Ang-(1–7) encapsulated in oligosaccharide hydroxypropyl-cyclodextrin (HPβCD) to protect the degradation of the peptide by enzymes in the digestive system. This oral Ang-(1–7) formulation has been tested in rats with myocardial infarction and shown to be cardioprotective [65]. Moreover, the same oral Ang-(1–7) formulation has reduced lung inflammation and histopathological changes of elastase induced pulmonary emphysema induced in mice [66]. A recent study in high fat diet fed rats with metabolic syndrome demonstrated that oral HPβCD/Ang-(1–7) treatment has the potential to decrease ACE and AT1R, increase ACE2 gene expression, reduce oxidative stress, and improve insulin signaling in the liver [67].

Another potential therapeutic target to treat liver fibrosis is ACE2. Increasing activity of ACE2 would be expected to facilitate the degradation of the profibrotic peptide Ang II while simultaneously enhancing the generation of the antifibrotic peptide Ang-(1–7). In experimental animal studies, recombinant human ACE2 (rhACE2) reduced hypertension in cardiovascular disease [68] and improved kidney function in diabetic nephropathy [69]. A phase I clinical trial has shown that rhACE2 was well tolerated in healthy human volunteers, without producing any unwanted side effects in the cardiovascular system [70]. However, adequately powered randomized clinical trials in healthy individuals and patients assigned for rhACE2 treatment are yet to be undertaken. There is one study that has tested the therapeutic effects of recombinant ACE2 in experimental liver fibrosis, using BDL and CCl_4_ models [71]. This study showed that recombinant ACE2 significantly reduced the liver fibrosis in both animal models [71]. Additionally, following CCl_4_ injection, ACE2 gene knockout (ACE2-KO) mice had high levels of α-SMA protein and collagen content in the liver compared with wild-type littermates [71]. These findings suggest that ACE2 is a potential therapy for liver fibrosis.

There are several disadvantages with systemic administration of recombinant ACE2. This includes the need for daily injections and expense, and the fact that circulating ACE2 could produce off target effects in other organs. Moreover, long-term therapy with systemic infusion of ACE2 may lead to systemic hypotension. 

The ideal approach to circumvent this problem would be to increase tissue-specific ACE2 levels in the target organ. To achieve this our group has developed an adeno-associated viral (AAV) vector system to deliver ACE2 into the liver of experimental animal models [36]. Although there are a number of viral vectors that have been used to date to increase expression of proteins, adeno-associated viral (AAV) vector appears to be the most safe and effective [72]. In literature, it has been shown that the AAV is an efficient vector to deliver a transgene and it provides many advantages compared to those of other viral vector candidates. The most prominent advantages of AAV over the other viral vectors include replicative defectiveness, nonpathogenicity, minimal immunogenicity, and broad tissue tropism in both animal models and humans. Moreover, the AAV gene delivery system has an ability to maintain long-term gene and protein expression and high tissue tropism following a single injection of the vector which further facilitates to its popularity as a better tool for gene delivery. This type of gene delivery system has been tested for inherited metabolic diseases [73]. 

An AAV vector carrying AAV2 genome and a liver-specific AAV8 capsid (AAV2/8) has been used to deliver murine ACE2 (mACE2) into the liver of three short-term experimental mouse models with liver disease [36]. This included a liver disease model induced by BDL (2-week model), toxin induced liver injury model by CCl_4_ injections (8-week model) and a dietary liver disease model induced by feeding MCD diet (8-week model), representing a cholestatic biliary fibrosis, alcoholic liver fibrosis and non-alcoholic fatty liver disease (NAFLD), respectively, in humans. It was shown that a single intraperitoneal injection of mACE2-rAAV2/8 therapy significantly reduced hepatic fibrosis in all three models. Furthermore, the treatment effects were confined to the liver for up to 6 months and, ACE2 overexpression was absent in other major organs such as heart, lungs, brain, intestine, and kidneys. ACE2 treated mice showed markedly increased ACE2 expression and activity in their livers and those results were accompanied by increased hepatic Ang-(1–7) levels with concomitant decrease in hepatic Ang II levels [36]. These findings provided profound evidence to conclude that liver-specific ACE2 gene delivery is a promising therapy for hepatic fibrosis. 

Recently, this treatment strategy has been tested and evaluated in a long-term animal model which better reflects the time course of liver disease in humans. This genetic model of biliary fibrosis is known as multiple drug resistant gene 2 knockout (Mdr2-KO) model and the biliary lesions seen in this chronic cholestatic disease are comparable to those seen in patients with primary sclerosing cholangitis (PSC). A study conducted to determine the therapeutic potential of mACE2-rAAV2/8 gene therapy at early and advanced biliary fibrosis of Mdr2-KO mice showed a marked attenuation in liver injury and fibrosis at both stages [64]. 

In recent years, it has been reported that the so-called ACE2 activator drugs also have potential to be used as therapeutic agents for tissue fibrosis. In 2007, Hernandez Prada and colleagues identified two small molecules, xanthenone (XNT) and resorcinolnaphthalein, as potential ACE2 activators using a structure based screening method [74]. These two compounds were tested in in vitro studies, and it was confirmed that they have the capacity to increase ACE2 activity in a dose dependent manner. However, only XNT was used in further testing in vivo as it was more soluble than resorcinolnaphthalein. This study showed that long term (4 weeks) administration of XNT could reverse myocardial, perivascular, and renal fibrosis in a spontaneously hypertensive rat (SHR) model. Later, other studies showed that XNT has a potential to increase ACE2 activity in thrombi of SHR with a significant antithrombogenic effect [75]. It was also shown that ACE2 activation by XNT protected experimental pregnant rats against leptin induced hypertension and proteinuria [76] and reduced hypertension-induced cardiac fibrosis in SHR [77]. Moreover, it was also shown to increase brain tissue ACE2 gene expression in cerebral ischemia/reperfusion injury in experimental rats [78]. 

In 2013, it was demonstrated that diminazene aceturate (DIZE), the US Food and Drug Administration (FDA) approved small molecule, was also effective as an ACE2 activator in rat models with ischemia induced cardiac disease and pulmonary hypertension [79,80]. DIZE has been developed to be used as an antitrypanosomal and antibabesial drug which has been used in veterinary practice for many decades [81]. The drug is used to treat human trypanosomiasis in some tropical and subtropical African countries [82]. In published studies, DIZE has been shown to exert beneficial effects on tissue fibrosis in the heart, lungs, kidneys, and the eyes [83,84,85]. A recent study published by our laboratory demonstrated however that DIZE inhibited experimental liver fibrosis but it did not affect liver ACE2 activity, questioning its mode of action [37]. 

### 1.4. The Role of RAS in Portal Hypertension

Almost 90% of the patients with cirrhosis eventually develop portal hypertension. This condition prequels to a number of complications that occur in the patients with decompensated cirrhosis, such as gastro-esophageal varices and variceal bleeding, ascites, hepato-renal syndrome (HRS), and hepato-pulmonary syndrome (HPS) [86,87,88,89]. 

In cirrhosis, the development portal hypertension is due to the combined effects of increased hepatic resistance to the incoming portal venous flow which results from the changes to the normal tissue architecture within the fibrotic liver and increased sinusoidal vasoconstriction, and elevated portal venous blood flow secondary to the splanchnic vasodilatation [90,91]. There is currently a lack of therapeutic options for treating portal hypertension and associated complications. The pharmacological mainstay is the use of non-selective beta-blockers (NSBBs). NSBBs reduce portal pressure via decreasing cardiac output and increasing splanchnic vascular resistance and thus reducing mesenteric blood flow. However, NSBBs are poorly tolerated thus contraindicated in up to 15–20% of the patients. Moreover, in up to 60% of the patients, NSBB treatment fails to produce a clinically significant therapeutic response, defined as a fall of hepatic venous pressure gradient of to a value less than 12 mmHg or reduction in portal pressure greater than 20% from baseline [92,93,94,95]. Since there have been no new drug classes introduced for the long-term management of portal hypertension for more than 30 years, there is an ongoing need for the development of more effective and tolerable drugs to treat this condition in cirrhotic patients. 

**Figure 4:**
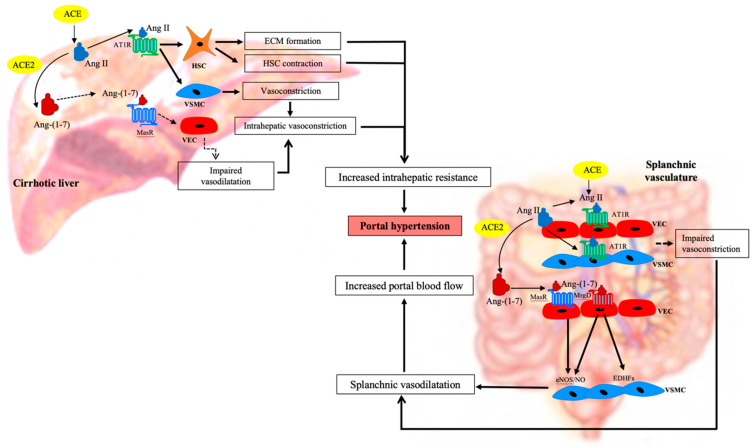
Role of the renin angiotensin system in cirrhotic portal hypertension. Development of portal hypertension in cirrhosis is a combined effect of the changes that occur within the intrahepatic and splanchnic vascular beds. In the cirrhotic liver, vasoconstrictor peptide angiotensin II (Ang II) signals through its receptor Ang II type 1 receptor (AT1R) in activated hepatic stellate cells (HSCs) to increase the extracellular matrix proteins (ECM) deposition, creating a fixed barrier to the incoming portal blood flow which raises portal pressure. In addition, Ang II also promotes the contraction of the activated HSCs and vascular smooth muscle cells (VSMCs), further increasing intrahepatic vascular tone, exacerbating portal pressure. Increased intrahepatic resistance is further augmented by the reduced release of vasodilatory molecules such as nitric oxide (NO) from vascular/sinusoidal endothelial cells (VECs) in the cirrhotic livers. In addition, the intrahepatic vasodilatory function of angiotensin-(1–7) (Ang-(1–7)) peptide produced from Ang II by ACE2 action is also diminished, further contributing to intrahepatic resistance. In contrast, in the cirrhotic splanchnic vascular bed, circulating Ang-(1–7) increases the release of NO via its putative receptor Mas (MasR) and possibly other factors such as endothelium-derived hyperpolarizing factors (EDHFs) via the Mas related G protein-coupled receptor type-D (MrgD) from VECs. This promotes the relaxation of VSMCs which leads to dilatation of the splanchnic vascular bed leading to an increased portal blood flow. This further aggravates portal pressure. Splanchnic vasodilatation is also aggravated by intrinsic splanchnic vascular hypocontractility to vasoconstrictors such as Ang II. ACE, angiotensin converting enzyme; ACE2, angiotensin converting enzyme 2; eNOS, endothelial nitric oxide synthase. Adapted from [102].

Recent studies suggest that the RAS plays a major role in the development of portal hypertension in cirrhosis [86,96,97,98] (Figure 4). Many experimental and clinical studies have shown that modulation of the RAS improves portal pressure in cirrhotic animal models and human patients [24,95,99,100,101], suggesting that this system is a potential target in formulating future therapies to treat portal hypertension in cirrhosis.

### 1.5. The Role of RAS in Increasing Hepatic Resistance in the Cirrhotic Liver

As outlined above, the components of the classical RAS, such as ACE and Ang II, are significantly upregulated in the systemic and hepatic circulations of the cirrhotic animals [35,39,48,63]. Ang II is a potent profibrotic peptide which promotes HSC proliferation and ECM formation in the fibrotic liver [31,39], and the subsequent elevation in intrahepatic resistance to blood flow contributes to the development of portal hypertension in cirrhosis. Increased intrahepatic vascular resistance is also mediated by the contraction of the activated perisinusoidal contractile HSCs. These cells overexpress the AT1R and contract in response to elevated Ang II, and it is through this mechanism that Ang II contributes to sinusoidal vasoconstriction, thus increasing the intrahepatic resistance to blood flow [32,41]. Previous work from our laboratory supported this concept by showing that in response the infusion of Ang II, perfused cirrhotic rat liver preparations had a greatly increased vasoconstrictive response when compared to healthy rat livers, likely due to the effects of Ang II on the upregulated AT1R in vascular smooth muscle cells and sinusoidal myofibroblastic HSCs [101]. This study also showed that cirrhotic livers have an elevated local Ang II production, thus potentially driving AT1R-mediated vasoconstriction and resistance to portal flow. Therefore, it is expected that the inhibition of Ang II via ACEi and/or ARBs would improve portal hypertension not only by attenuating hepatic fibrosis but also by improving intrahepatic resistance to blood flow in cirrhosis [48,103,104].

Recent studies suggest that the detrimental effects of the classical RAS on hepatic resistance could be counteracted by increasing the overexpression of the alternate RAS in the cirrhotic livers [3]. Previous work by our laboratory showed that the addition of Ang-(1–7) or the MasR agonist AVE0991, decreases the activation of primary rat HSCs in cell culture, whereas with the addition of MasR blocker A779, increases the activation of HSC, as reflected by increased αSMA expression. This study also showed that infusion of Ang-(1–7) in-vivo, suppressed HSC activation in cirrhotic rats [32]. In keeping with these findings, a separate study published by our group showed that Ang-(1–7) has profound vasodilatory effects in cirrhotic livers [24]. In this study in-situ perfused cirrhotic rat livers pre-constricted with Ang II or methoxamine, produced a marked vasodilatory response to the infusion of Ang-(1–7) infusion of Ang-(1–7), which is also shown to be endothelium-dependent and AT1R/AT2R independent [24]. In addition, the local overexpression of ACE2 in cirrhosis [36] not only ameliorated liver fibrosis but also reduced hepatic perfusion pressure in experimental mouse models by decreasing the levels of vasoconstrictor peptide Ang II whilst simultaneously increasing the levels of vasodilator peptide Ang-(1–7) in the cirrhotic livers [36,64].

### 1.6. The Role of RAS in Mesenteric Vasodilatation in Cirrhosis

In contrast to the profound vasoconstrictive effects produced by Ang II in the intrahepatic vasculature, systemic and splanchnic vessels are hyporesponsive to circulatory Ang II, and therefore these vessels remain dilated in cirrhosis [105,106,107]. Experiments with the isolated mesenteric, omental and/or peripheral vascular bed preparations from cirrhotic animals and patients have indicated that the dilatation of the splanchnic vascular bed in cirrhosis is also attributed to the development of intrinsic vascular hyporesponsiveness to the circulatory vasoconstrictors such as Ang II [108,109]. Supporting the above in-vitro vessel work, in-vivo experiments in human patients showed that cirrhotic patients had a diminished vasoconstrictive response to intra-arterially administered Ang II as reflected by no significant change in the forearm blood flow, compared to healthy controls [110]. However, despite the evidence of reduced Ang II activity, AT1R is either unchanged or upregulated in splanchnic vessels of the cirrhotic patients [111,112], suggesting that splanchnic vascular hyporesponsiveness to Ang II may probably as a result of the changes that occur downstream of the AT1R [113,114]. 

Systemic vasodilatation in cirrhosis may also be facilitated by overexpression of the alternate RAS [32,35,115]. Importantly, ACE2, MasR and Ang-(1–7) are elevated in the splanchnic vascular bed of cirrhotic rats and human patients, suggesting that the alternate RAS may play a role in the splanchnic vasodilatation and thereby in the development of portal hypertension in cirrhosis [99,100]. Ang-(1–7) acting via the MasR is shown to increase the release of nitric oxide (NO) from the VECs, which promotes the relaxation of VSMCs leading to splanchnic vasodilatation [99]. It is also shown that Ang-(1–7)/Ang II ratio is significantly elevated in the splanchnic compared to systemic circulations in the cirrhotic patients undergoing liver transplant, which is also negatively correlated with the systemic vascular resistance, suggesting that the augmented Ang-(1–7) activity contributes to the splanchnic vasodilatation in cirrhosis [115]. Supporting this, a previous study by our group showed that the infusion of Ang-(1–7) produced hypocontractility in the cirrhotic splanchnic vasculature but not in controls [99]. Consistent with this, preincubation of cirrhotic mesenteric vascular bed preparations with MasR blocker A779 inhibited the vasodilatory effects produced by Ang-(1–7) [99]. These ex vivo findings were in agreement with in vivo findings showing that a bolus intravenous injection of Ang-(1–7) reduced both splanchnic vascular resistance and hepatic vascular resistance and that a bolus dose of the MasR antagonist A779 increased the resistance in both splanchnic and hepatic vascular beds with a net effect of a significant improvement of portal pressure in CCl_4_-induced cirrhotic rats [99].

In our recent experiments we were the first to document that not only the MasR, but the alternate receptor for Ang-(1–7), MrgD, is also upregulated in the splanchnic vessels of the cirrhotic rats. Moreover, the injection of both MasR and MrgD blockers improved portal hypertension in cirrhotic animal models, presumably via the inhibition of Ang-(1–7) mediated splanchnic vasodilatation in cirrhosis [100]. Although this study did not investigate the mechanism(s) through which Ang-(1–7) regulates the MrgD-mediated vasodilatory effects in the splanchnic vessels, it is possible that in addition to the release of NO, Ang-(1–7), by acting via the MrgD, may enhance the release of other vasodilators such as endothelium-derived hyperpolarizing factors (EDHFs) including epoxyeicosatrienoic acids in splanchnic vessels [116,117].

### 1.7. Manipulation of the RAS in Portal Hypertension

Thus, much research evidence supports the contribution of the RAS in the pathogenesis of portal hypertension. It is expected that the inhibition of the classical RAS or increasing local expression or activity of the alternate RAS may reduce intrahepatic vascular tone, resulting in a reduced portal pressure. On the other hand, inhibition of the alternate RAS in the splanchnic vasculature is expected to increase splanchnic vascular resistance, thereby improving portal hypertension by reducing portal inflow. These findings therefore suggest that both the classical and alternate RAS are potential targets for the development of novel therapies to treat portal hypertension in human cirrhotic patients.

### 1.8. Targeting the Classical RAS in Portal Hypertension

Many experimental and clinical studies have shown that portal hypertension in cirrhosis could be treated with the inhibitors of the classical RAS, such as ACEi and ARBs. In addition to RAS regulation of portal hypertension, some studies have also suggested that chymase inhibitors are potential drugs to reduce portal pressure by inhibiting intrahepatic Ang II production [10,11]. These drugs are expected to reduce portal pressure via decreasing Ang II mediated increase in intrahepatic resistance. ACEi also prevents the degradation of Ang-(1–7), and thus would increase the intrahepatic Ang-(1–7) levels promoting vasodilatation. However, although they have been widely used in the treatment of systemic hypertension, the ACEi such as enalapril and captopril, and ARBs such as candesartan, losartan, and irbesartan have only been used in a limited number of clinical trials to study their antifibrotic and antiportal hypertensive effects in cirrhotic patients [111,118,119,120,121,122].

The outcomes of clinical trials studying the effects of ACEi and ARBs in cirrhosis were summarized by Tandon and colleagues in a comprehensive meta-analysis published in 2010, which included the findings of three and nine studies on ACEi ARBs, respectively [95]. This analysis concluded that in early Child Pugh A cirrhosis, ACEi and/or ARBs are of similar efficacy to NSBBs in reducing the hepatic venous pressure gradient (HVPG) (17% and 21% with ACEi/ARBs and NSBBs, respectively). However, in advanced Child Pugh B or C cirrhosis, ACEi/ARBs only produced a 3% reduction of HVPG, whilst NSBBs produced a similar reduction of HVPG to that in Child Pugh A cirrhosis. This study, therefore, concluded that although the RAS plays an important role in increasing intrahepatic resistance in early stages of cirrhosis, the effects of RAS on increasing intrahepatic vascular tone in advanced cirrhosis are likely to be overridden by the activation of other powerful vasoconstrictive systems such as the endothelin and/or the sympathetic nervous system [95,107,123]. However, an undesirable side effect of ACEi/ARBs is that in patients with advanced cirrhosis these drugs produce significant systemic hypotension and renal impairment, since the baseline activation of the RAS plays a pivotal role in maintaining adequate arterial pressure and renal perfusion in these patients [95,124,125]. 

### 1.9. Targeting the Alternate RAS in Portal Hypertension

It is shown that the vasodilatory effects of Ang-(1–7) in cirrhotic splanchnic vessels appear to be mediated via its receptor MasR. Administration of the specific MasR blocker A779 increased the resistance in splanchnic vessels, reduced splanchnic blood flow and thereby improved portal hypertension in cirrhotic rat models [99]. However, the effectiveness of MasR blockade in lowering portal pressure by increasing splanchnic resistance may be compromised by its ability to increase intrahepatic resistance by blocking Ang-(1–7) mediated vasodilatation in the liver [99]. Indeed, in contrast to the results of MasR antagonism, the nonpeptide Ang-(1–7) agonist AVE0991 was shown to lower portal pressure by reducing intrahepatic resistance [126]. Supporting this another study showed that neutral endopeptidase (NEP) inhibitor candoxatrilat also significantly reduced intrahepatic resistance thereby portal pressure in cirrhotic rats, via reducing Ang-(1–7) metabolism in the cirrhotic livers [127].

**Figure 5:**
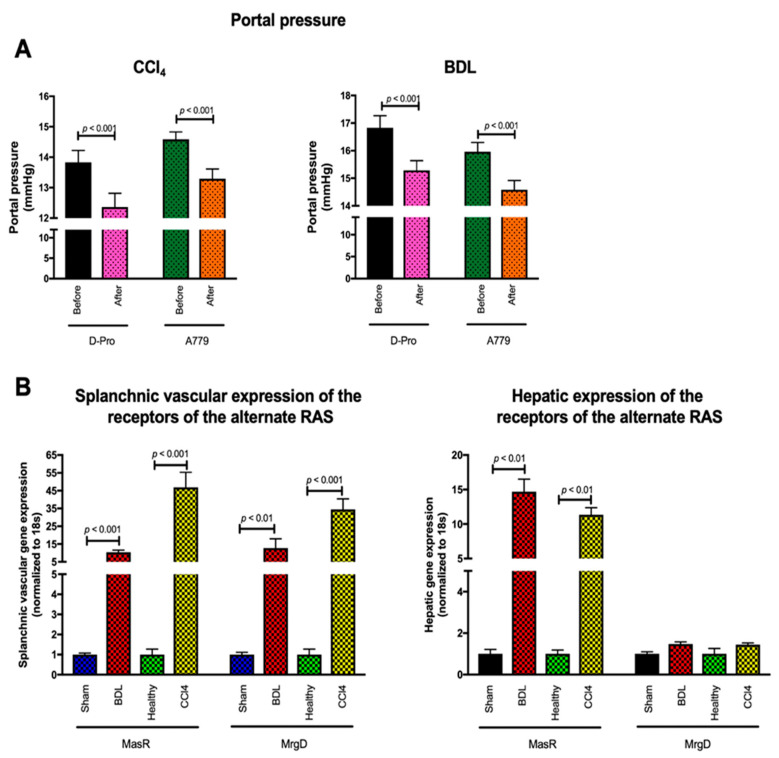
Portal pressure responses to the blockade of the receptors of the alternate renin angiotensin system (RAS) in cirrhotic rat models induced by carbon tetrachloride (CCl_4_) injections or bile duct ligation (BDL), and splanchnic and hepatic vascular expression of the receptors, MasR and Mas related G protein-coupled receptor type-D (MrgD). (**A**) Portal pressure responses 5 min after a bolus injection of MasR blocker D-Ala7-Ang-(1–7) (A779) (10 µg/kg) or MrgD blocker D-Pro7-Ang-(1–7) (D-Pro) (10 µg/kg) in CCl4 and BDL rats. Both MasR and MrgD blockade produced a significant reduction of portal pressure likely via blocking angiotensin-(1–7)-mediated dilatation of the splanchnic vascular bed. (**B**) Gene expression of MasR and MrgD in cirrhotic mesenteric and hepatic vascular beds of CCl4 and BDL models compared with sham-operated or healthy control livers. In the splanchnic vasculature of cirrhotic rats, both MasR and MrgD are upregulated, suggesting that both these receptors likely play an important role in angiotensin-(1–7)-mediated splanchnic vasodilatation in cirrhosis. Although the MasR is upregulated in the cirrhotic livers, there was no change in the expression of MrgD suggesting that MasR, but not MrgD contributes to the regulation of hepatic vascular resistance in cirrhosis. Adapted from [100].

As has been discussed, in addition to the MasR, the novel receptor MrgD is also shown to mediate the vasodilatory effects of Ang-(1–7) [25]. Consistent with this, we have documented that similar to the MasR, MrgD is also significantly upregulated in the cirrhotic splanchnic vessels, and blockade of MrgD with a bolus injection of D-Pro led to a significant reduction of portal pressure in cirrhotic rat models, which was similar to that produced by the MasR blocker A779 (Figure 5A) [100]. However, in this study both these blockers failed to reduce portal pressure up to clinically significant levels (i.e., <20% from the baseline). Moreover, these drugs failed to sustain their portal pressure lowering effect for more than 20–25 min, possibly attributed to the rapid metabolism of these peptide blockers within the rat circulation [100]. This study therefore warrants further studies to determine whether these blockers could maintain adequate plasma concentrations when given as a continuous infusion over time, and thereby produce a clinically sustainable effect on portal pressure in experimental cirrhosis. 

Importantly, we have also discovered that unlike MasR, MrgD is not upregulated in the hepatic vascular bed of the cirrhotic animals (Figure 5B), suggesting that the effects of MrgD likely to be limited to the splanchnic vascular bed in cirrhosis. Thus, in contrast to the MasR blocker A779, MrgD blockade with D-Pro may not increase intrahepatic resistance, thus enhancing its antiportal hypertensive effect in cirrhosis [99]. This finding that the MrgD has tissue-specific expression in splanchnic vessels in cirrhosis has significant potential implications for the development of pharmacotherapies that specifically target splanchnic vasodilatation in cirrhotic patients. 

## 2. Conclusions

Recent advances in our understanding of the complexities of the RAS have led to the development of new ideas regarding the therapeutic potential of manipulating the RAS in liver disease. Whilst the classical RAS via its effector peptide Ang II is strongly implicated in liver scarring, there remains a lack of clinical evidence to support the routine use of classical RAS blockers as antifibrotic agents. Ang II is also strongly implicated in the pathogenesis of portal hypertension via its ability to promote constriction of contractile cells in the cirrhotic liver. This has prompted a number of clinical trials of classical RAS blockers in cirrhosis; however, available evidence suggests that this class of drugs is of limited efficacy and has serious adverse effects in patients with advanced cirrhosis. The possible role of the alternative RAS and its suitability as a therapeutic target in liver cirrhosis is less well-studied. There is intriguing evidence that drugs targeting the MasR could have efficacy as antifibrotic agents. On the other hand, a greater therapeutic interest and importance is the possibility that drugs targeting the receptors of the alternate arm could be used to treat portal hypertension. Very recent evidence suggests that the MrgD may be a particularly attractive therapeutic target because in cirrhosis, unlike MasR, the expression of MrgD is minimal in the liver but markedly upregulated in splanchnic vessels. This could allow for the potential development of mesenteric vasculature selective drugs which reduce splanchnic flow but do not adversely affect resistance and blood flow in the liver.

## References

[1] Friedman S.L. (2008). Mechanisms of hepatic fibrogenesis. Gastroenterology.

[2] Schuppan D., Afdhal N.H. (2008). Liver cirrhosis. Lancet.

[3] Grace J.A., Herath C.B., Mak K.Y., Burrell L.M., Angus P.W. (2012). Update on new aspects of the renin–angiotensin system in liver disease: Clinical implications and new therapeutic options. Clin. Sci..

[4] WHO (2019). Summary Tables of Mortality Estimates by Cause, Age and Sex, Globally and by Region, 2000–2016.

[5] Zhou W.-C., Zhang Q.-B., Qiao L. (2014). Pathogenesis of liver cirrhosis. World J. Gastroenterol..

[6] Rajapaksha I.G., Angus P.W., Herath C.B. (2019). Current therapies and novel approaches for biliary diseases. World J. Gastrointest. Pathophysiol..

[7] Berzigotti A. (2017). Advances and challenges in cirrhosis and portal hypertension. BMC Med..

[8] Warner F.J., Lubel J.S., McCaughan G.W., Angus P.W. (2007). Liver fibrosis: A balance of ACEs?. Clin. Sci..

[9] Lubel J.S., Herath C.B., Burrell L.M., Angus J.A. (2008). Liver disease and the renin-angiotensin system: Recent discoveries and clinical implications. J. Gastroenterol. Hepatol..

[10] Sansoè G., Aragno M., Mastrocola R., Mengozzi G., Novo E., Parola M. (2016). Role of Chymase in the Development of Liver Cirrhosis and Its Complications: Experimental and Human Data. PLoS ONE.

[11] Komeda K., Takai S., Jin D., Tashiro K., Hayashi M., Tanigawa N., Miyazaki M. (2010). Chymase inhibition attenuates tetrachloride-induced liver fibrosis in hamsters. Hepatol. Res..

[12] Steckelings U.M., Larhed M., Hallberg A., E Widdop R., Jones E.S., Wallinder C., Namsolleck P., Dahlöf B., Unger T. (2011). Non-peptide AT2-receptor agonists. Curr. Opin. Pharmacol..

[13] De Macêdo S.M., Guimarães T.A., Feltenberger J.D., Santos S.H.S. (2014). The role of renin-angiotensin system modulation on treatment and prevention of liver diseases. Peptides.

[14] Prasad A., A Quyyumi A. (2004). Renin-Angiotensin System and Angiotensin Receptor Blockers in the Metabolic Syndrome. Circulation.

[15] Donoghue M., Hsieh F., Baronas E., Godbout K., Gosselin M., Stagliano N., Donovan M., Woolf B., Robison K., Jeyaseelan R. (2000). A Novel Angiotensin-Converting Enzyme–Related Carboxypeptidase (ACE2) Converts Angiotensin I to Angiotensin 1-9. Circ. Res..

[16] Tipnis S.R., Hooper N.M., Hyde R., Karran E., Christie G., Turner A.J. (2000). A Human Homolog of Angiotensin-converting Enzyme: Cloning and Functional Expression as a Captopril-Insensitive Carboxypeptidase. J. Biol. Chem..

[17] Schiavone M.T., Santos R.A., Brosnihan K.B., Khosla M.C., Ferrario C.M. (1988). Release of vasopressin from the rat hypothalamo-neurohypophysial system by angiotensin-(1–7) heptapeptide. Proc. Natl. Acad. Sci. USA.

[18] Chappell M.C. (2019). The Angiotensin-(1–7) Axis: Formation and Metabolism Pathways. Angiotensin-(1–7).

[19] Santos R.A., e Silva A.C.S., Maric C., Silva D.M., Machado R.P., de Buhr I., Heringer-Walther S., Pinheiro S.V.B., Lopes M.T., Bader M. (2003). Angiotensin-(1–7) is an endogenous ligand for the G protein-coupled receptor Mas. Proc. Natl. Acad. Sci. USA.

[20] Santos R.A., Haibara A.S., Campagnole-Santos M.J., Simões e Silva A.C., Paula R.D., Pinheiro S.V., de Fátima Leite M., Lemos V.S., Silva D.M., Guerra M.T. (2003). Characterization of a New Selective Antagonist for Angiotensin-(1–7), d-Pro^7^-Angiotensin-(1–7). Hypertension.

[21] Silva D., Vianna H., Cortes S., Campagnole-Santos M., Santos R., Lemos V.S. (2007). Evidence for a new angiotensin-(1–7) receptor subtype in the aorta of Sprague–Dawley rats. Peptides.

[22] Gembardt F., Grajewski S., Vahl M., Schultheiss H.-P., Walther T. (2008). Angiotensin metabolites can stimulate receptors of the Mas-related genes family. Mol. Cell. Biochem..

[23] Lautner R.Q., Villela D.C., Fraga-Silva R.A., Silva N., Verano-Braga T., Costa-Fraga F., Jankowski J., Jankowski V., Sousa F., Alzamora A. (2013). Discovery and characterization of alamandine: A novel component of the renin-angiotensin system. Circ. Res..

[24] Herath C.B., Mak K., Burrell L.M., Angus P.W. (2013). Angiotensin-(1–7) reduces the perfusion pressure response to angiotensin II and methoxamine via an endothelial nitric oxide-mediated pathway in cirrhotic rat liver. Am. J. Physiol. Liver Physiol..

[25] Tetzner A., Gebolys K., Meinert C., Klein S., Uhlich A., Trebicka J., Villacañas Ó., Walther T. (2016). G-Protein–Coupled Receptor MrgD Is a Receptor for Angiotensin-(1–7) Involving Adenylyl Cyclase, cAMP, and Phosphokinase. Hypertension.

[26] Bader M. (2010). Tissue Renin-Angiotensin-Aldosterone Systems: Targets for Pharmacological Therapy. Annu. Rev. Pharmacol. Toxicol..

[27] Nehme A., Zouein F.A., Zayeri Z.D., Zibara K. (2019). An Update on the Tissue Renin Angiotensin System and Its Role in Physiology and Pathology. J. Cardiovasc. Dev. Dis..

[28] Bataller R., Sancho-Bru P., Gines P., Lora J.M., Al-Garawi A., Solé M., Colmenero J., Nicolás J.M., Jiménez W., Weich N. (2003). Activated human hepatic stellate cells express the renin-angiotensin system and synthesize angiotensin II. Gastroenterology.

[29] Lang C.C., Struthers A.D. (2013). Targeting the renin-angiotensin-aldosterone system in heart failure. Nat. Rev. Cardiol..

[30] Nistala R., Wei Y., Sowers J.R., Whaley-Connell A. (2009). Renin-angiotensin-aldosterone system-mediated redox effects in chronic kidney disease. Transl. Res..

[31] Bataller R., Schwabe R.F., Choi Y.H., Yang L., Paik Y.H., Lindquist J., Qian T., Schoonhoven R., Hagedorn C.H., Lemasters J.J. (2003). NADPH oxidase signal transduces angiotensin II in hepatic stellate cells and is critical in hepatic fibrosis. J. Clin. Investig..

[32] Lubel J.S., Herath C.B., Tchongue J., Grace J., Jia Z., Spencer K., Casley D., Crowley P., Sievert W., Burrell L.M. (2009). Angiotensin-(1–7), an alternative metabolite of the renin–angiotensin system, is up-regulated in human liver disease and has antifibrotic activity in the bile-duct-ligated rat. Clin. Sci..

[33] Paizis G., Cooper M.E., Schembri J.M., Tikellis C., Burrell L.M., Angus P.W. (2002). Up-regulation of components of the renin-angiotensin system in the bile duct–ligated rat liver. Gastroenterology.

[34] Paul M., Mehr A.P., Kreutz R. (2006). Physiology of Local Renin-Angiotensin Systems. Physiol. Rev..

[35] Herath C.B., Warner F.J., Lubel J.S., Dean R.G., Jia Z., Lew R.A., Smith A.I., Burrell L.M., Angus J.A. (2007). Upregulation of hepatic angiotensin-converting enzyme 2 (ACE2) and angiotensin-(1–7) levels in experimental biliary fibrosis. J. Hepatol..

[36] Mak K.Y., Chin R., Cunningham S.C., Habib M.R., Torresi J., Sharland A.F., E Alexander I., Angus J.A., Herath C.B. (2015). ACE2 Therapy Using Adeno-associated Viral Vector Inhibits Liver Fibrosis in Mice. Mol. Ther..

[37] Rajapaksha I.G., Mak K.Y., Huang P., Burrell L.M., Angus J.A., Herath C.B. (2018). The small molecule drug diminazene aceturate inhibits liver injury and biliary fibrosis in mice. Sci. Rep..

[38] Friedman S.L. (2008). Hepatic Stellate Cells: Protean, Multifunctional, and Enigmatic Cells of the Liver. Physiol. Rev..

[39] Bataller R., Gäbele E., Parsons C.J., Morris T., Yang L., Schoonhoven R., A Brenner D., Rippe R.A. (2005). Systemic infusion of angiotensin II exacerbates liver fibrosis in bile duct-ligated rats. Hepatology.

[40] Herath C.B., Mak K.Y., Angus P.W. (2015). Role of the Alternate RAS in Liver Disease and the GI Tract. The Protective Arm of the Renin Angiotensin System (RAS).

[41] Bataller R., Ginès P., Nicolás J.M., Görbig M., Garcia–Ramallo E., Gasull X., Bosch J., Arroyo V., Rodés J. (2000). Angiotensin II induces contraction and proliferation of human hepatic stellate cells. Gastroenterology.

[42] Deleve L.D. (2015). Liver sinusoidal endothelial cells in hepatic fibrosis. Hepatology.

[43] Maretti-Mira A.C., Wang X., Wang L., DeLeve L.D. (2016). Role of incomplete stem cell maturation in hepatic fibrosis. HEPATOLOGY.

[44] Jarnagin W.R., Rockey D.C., E Koteliansky V., Wang S.S., Bissell D.M. (1994). Expression of variant fibronectins in wound healing: Cellular source and biological activity of the EIIIA segment in rat hepatic fibrogenesis. J. Cell Biol..

[45] Marrone G., Shah V.H., Gracia-Sancho J. (2016). Sinusoidal communication in liver fibrosis and regeneration. J. Hepatol..

[46] Jonsson J.R., Clouston A.D., Ando Y., Kelemen L.I., Horn M.J., Adamson M.D., Purdie D.M., Powell E.E. (2001). Angiotensin-Converting Enzyme Inhibition Attenuates the Progression of Rat Hepatic Fibrosis. Gastroenterology.

[47] Turkay C., Yönem Ö., Arıcı S., Koyuncu A., Kanbay M., Arici S. (2007). Effect of Angiotensin-converting Enzyme Inhibition on Experimental Hepatic Fibrogenesis. Dig. Dis. Sci..

[48] Paizis G., Gilbert R.E., Cooper M.E., Murthi P., Schembri J.M., Wu L.L., Rumble J.R., Kelly D.J., Tikellis C., Cox A. (2001). Effect of angiotensin II type 1 receptor blockade on experimental hepatic fibrogenesis. J. Hepatol..

[49] Yi E.-T., Liu R.-X., Wen Y., Yin C.-H. (2012). Telmisartan attenuates hepatic fibrosis in bile duct-ligated rats. Acta Pharmacol. Sin..

[50] Wei H.-S., Li D.-G., Lu H.-M., Zhan Y.-T., Wang Z.-R., Huang X., Zhang J., Cheng J.-L., Xu Q.-F. (2000). Effects of AT1 receptor antagonist, losartan, on rat hepatic fibrosis induced by CCl4. World J. Gastroenterol..

[51] Hirose A., Ono M., Saibara T., Nozaki Y., Masuda K., Yoshioka A., Takahashi M., Akisawa N., Iwasaki S., Oben J.A. (2007). Angiotensin II type 1 receptor blocker inhibits fibrosis in rat nonalcoholic steatohepatitis. Hepatology.

[52] Corey K.E., Shah N., Misdraji J., Abu Dayyeh B.K., Zheng H., Bhan A.K., Chung R.T. (2009). The effect of angiotensin-blocking agents on liver fibrosis in patients with hepatitis C. Liver Int..

[53] Stokkeland K., Lageborn C.T., Höijer J., Bottai M., Stål P., Söderberg-Löfdal K., Ekbom A. (2018). Statins and Angiotensin-Converting Enzyme Inhibitors are Associated with Reduced Mortality and Morbidity in Chronic Liver Disease. Basic Clin. Pharmacol. Toxicol..

[54] Kim M.Y., Cho M.Y., Baik S.K., Jeong P.H., Suk K.T., Jang Y.O., Yea C.J., Kim J.W., Kim H.S., Kwon S.O. (2012). Beneficial effects of candesartan, an angiotensin-blocking agent, on compensated alcoholic liver fibrosis—A randomized open-label controlled study. Liver Int..

[55] Hidaka H., Nakazawa T., Shibuya A., Minamino T., Takada J., Tanaka Y., Okuwaki Y., Watanabe M., Koizumi W. (2011). Effects of 1-year administration of olmesartan on portal pressure and TGF-beta1 in selected patients with cirrhosis: A randomized controlled trial. J. Gastroenterol..

[56] Debernardi-Venon W., Martini S., Biasi F., Vizio B., Termine A., Poli G., Brunello F., Alessandria C., Bonardi R., Saracco G. (2007). AT1 receptor antagonist Candesartan in selected cirrhotic patients: Effect on portal pressure and liver fibrosis markers. J. Hepatol..

[57] Yoshiji H., Noguchi R., Ikenaka Y., Kaji K., Douhara A., Yamao J., Toyohara M., Mitoro A., Sawai M., Yoshida M. (2011). Combination of branched-chain amino acid and angiotensin-converting enzyme inhibitor improves liver fibrosis progression in patients with cirrhosis. Mol. Med. Rep..

[58] Terui Y., Saito T., Watanabe H., Togashi H., Kawata S., Kamada Y., Sakuta S. (2002). Effect of angiotensin receptor antagonist on liver fibrosis in early stages of chronic hepatitis C. Hepatology.

[59] McPherson S., Wilkinson N., Tiniakos D., Wilkinson J., Burt A.D., McColl E., Stocken D.D., Steen N., Barnes J., Goudie N. (2017). A randomised controlled trial of losartan as an anti-fibrotic agent in non-alcoholic steatohepatitis. PLoS ONE.

[60] Zhu Q., Li N., Li F., Zhou Z., Han Q., Lv Y., Sang J., Liu Z. (2016). Therapeutic effect of renin angiotensin system inhibitors on liver fibrosis. J. Renin-Angiotensin-Aldosterone Syst..

[61] Runyon B.A. (2013). Introduction to the revised American Association for the Study of Liver Diseases Practice Guideline management of adult patients with ascites due to cirrhosis 2012. Hepatology.

[62] EASL (2018). EASL Clinical Practice Guidelines for the management of patients with decompensated cirrhosis. J. Hepatol..

[63] Paizis G., Tikellis C., E Cooper M., Schembri J.M., A Lew R., I Smith A., Shaw T., Warner F.J., Zuilli A., Burrell L.M. (2005). Chronic liver injury in rats and humans upregulates the novel enzyme angiotensin converting enzyme 2. Gut.

[64] Rajapaksha I.G., Gunarathne L.S., Asadi K., Cunningham S.C., Sharland A., Alexander I.E., Angus P.W., Herath C.B. (2019). Liver-Targeted Angiotensin Converting Enzyme 2 Therapy Inhibits Chronic Biliary Fibrosis in Multiple Drug-Resistant Gene 2-Knockout Mice. Hepatol. Commun..

[65] Marques F.D., Ferreira A.J., Sinisterra R.D., Jacoby B.A., Sousa F.B., Caliari M.V., Silva G.A., Melo M.B., Nadu A.P., Souza L.E. (2011). An Oral Formulation of Angiotensin-(1–7) Produces Cardioprotective Effects in Infarcted and Isoproterenol-Treated Rats. Hypertension.

[66] Bastos A.C., Magalhães G.S., Gregório J.F., Matos N.A., Motta-Santos D., Bezerra F.S., Santos R.A.S., Santos M.J.C., Rodrigues-Machado M.G. (2020). Oral formulation angiotensin-(1–7) therapy attenuates pulmonary and systemic damage in mice with emphysema induced by elastase. Immunobiology.

[67] Figueiredo V.P., Barbosa M.A., De Castro U.G.M., Zacarias A.C., Bezerra F.S., De Sá R.G., De Lima W.G., Dos Santos R.A.S., Alzamora A.C. (2019). Antioxidant Effects of Oral Ang-(1–7) Restore Insulin Pathway and RAS Components Ameliorating Cardiometabolic Disturbances in Rats. Oxidative Med. Cell. Longev..

[68] Wysocki J., Ye M., Rodriguez E., González-Pacheco F.R., Barrios C., Evora K., Schuster M., Loibner H., Brosnihan K.B., Ferrario C.M. (2010). Targeting the Degradation of Angiotensin II With Recombinant Angiotensin-Converting Enzyme 2. Hypertension.

[69] Oudit G.Y., Liu G.C., Zhong J., Basu R., Chow F.L., Zhou J., Loibner H., Janzek E., Schuster M., Penninger J.M. (2009). Human Recombinant ACE2 Reduces the Progression of Diabetic Nephropathy. Diabetes.

[70] Haschke M., Schuster M., Poglitsch M., Loibner H., Salzberg M., Bruggisser M., Penninger J., Krähenbühl S. (2013). Pharmacokinetics and Pharmacodynamics of Recombinant Human Angiotensin-Converting Enzyme 2 in Healthy Human Subjects. Clin. Pharmacokinet..

[71] Österreicher C.H., Taura K., De Minicis S., Seki E., Penz-Österreicher M., Kodama Y., Kluwe J., Schuster M., Oudit G.Y., Penninger J.M. (2009). Angiotensin-converting-enzyme 2 inhibits liver fibrosis in mice. Hepatology.

[72] Naso M.F., Tomkowicz B., Perry W.L., Strohl W.R. (2017). Adeno-Associated Virus (AAV) as a Vector for Gene Therapy. BioDrugs.

[73] Alexander I.E., Cunningham S.C., Logan G.J., Christodoulou J. (2008). Potential of AAV vectors in the treatment of metabolic disease. Gene Ther..

[74] Prada J.A.H., Ferreira A.J., Katovich M.J., Shenoy V., Qi Y., Santos R.A.S., Castellano R.K., Lampkins A.J., Gubala V., Ostrov D.A. (2008). Structure-Based Identification of Small-Molecule Angiotensin-Converting Enzyme 2 Activators as Novel Antihypertensive Agents. Hypertension.

[75] Fraga-Silva R.A., Sorg B.S., Wankhede M., DeDeugd C., Jun J.Y., Baker M.B., Li Y., Castellano R.K., Katovich M.J., Raizada M.K. (2010). ACE2 Activation Promotes Antithrombotic Activity. Mol. Med..

[76] Ibrahim H.S., Froemming G.R.A., Omar E., Singh H.J. (2014). ACE2 activation by xanthenone prevents leptin-induced increases in blood pressure and proteinuria during pregnancy in Sprague-Dawley rats. Reprod. Toxicol..

[77] Ferreira A.J., Shenoy V., Qi Y., Fraga-Silva R.A., Santos R.A., Katovich M.J., Raizada M.K. (2011). Angiotensin-converting enzyme 2 activation protects against hypertension-induced cardiac fibrosis involving extracellular signal-regulated kinases. Exp. Physiol..

[78] Abdel-Fattah M.M., Messiha B.A.S., Mansour A.M. (2018). Modulation of brain ACE and ACE2 may be a promising protective strategy against cerebral ischemia/reperfusion injury: An experimental trial in rats. Naunyn-Schmiedeberg’s Arch. Pharmacol..

[79] Qi Y., Zhang J., Cole-Jeffrey C.T., Shenoy V., Espejo A., Hanna M., Song C., Pepine C.J., Katovich M.J., Raizada M.K. (2013). Diminazene Aceturate Enhances Angiotensin-Converting Enzyme 2 Activity and Attenuates Ischemia-Induced Cardiac Pathophysiology. Hypertension.

[80] Shenoy V., Gjymishka A., Jarajapu Y.P., Qi Y., Afzal A., Rigatto K., Ferreira A.J., Fraga-Silva R.A., Kearns P., Douglas J.Y. (2013). Diminazene Attenuates Pulmonary Hypertension and Improves Angiogenic Progenitor Cell Functions in Experimental Models. Am. J. Respir. Crit. Care Med..

[81] Peregrine A., Mamman M. (1993). Pharmacology of diminazene: A review. Acta Trop..

[82] Abaru D.E., Liwo D.A., Isakina D., Okori E.E. (1984). Retrospective long-term study of effects of berenil by follow-up of patients treated since 1965. Tropenmedizin und Parasitol..

[83] Qiu Y., Shil P.K., Zhu P., Yang H., Verma A., Lei B., Li Q. (2014). Angiotensin-Converting Enzyme 2 (ACE2) Activator Diminazene Aceturate Ameliorates Endotoxin-Induced Uveitis in Mice. Investig. Opthalmology Vis. Sci..

[84] Foureaux G., Nogueira J.C., Nogueira B.S., Fulgêncio G.O., Menezes G.B., Fernandes S.O.A., Cardoso V.N., Fernandes R.S., Oliveira G.P., Franca J.R. (2013). Antiglaucomatous Effects of the Activation of Intrinsic Angiotensin-Converting Enzyme 2. Investig. Opthalmology Vis. Sci..

[85] Tao L., Qiu Y., Fu X., Lin R., Lei C., Wang J., Lei B. (2016). Angiotensin-converting enzyme 2 activator diminazene aceturate prevents lipopolysaccharide-induced inflammation by inhibiting MAPK and NF-ΚB pathways in human retinal pigment epithelium. J. Neuroinflamm..

[86] Bosch J., Garcia-Pagan J.C. (2000). Complications of cirrhosis. I. Portal hypertension. J. Hepatol..

[87] Tandon P., Garcia-Tsao G. (2006). Portal Hypertension and Hepatocellular Carcinoma: Prognosis and Beyond. Clin. Gastroenterol. Hepatol..

[88] Abraldes J.G., Bosch J., Abraldeṣ J.G. (2007). Clinical Features and Natural History of Variceal Hemorrhage. Clinical Gastroenterology.

[89] Ripoll C., Groszmann R., Garcia–Tsao G., Grace N., Burroughs A., Planas R., Escorsell A., Garcia–Pagan J.C., Makuch R., Patch D. (2007). Hepatic Venous Pressure Gradient Predicts Clinical Decompensation in Patients With Compensated Cirrhosis. Gastroenterology.

[90] Martell M., Coll M., Ezkurdia N., Raurell I., Genescà J. (2010). Physiopathology of splanchnic vasodilation in portal hypertension. World J. Hepatol..

[91] Groszmann R.J., Abraldes J.G. (2005). Portal hypertension: From bedside to bench. J. Clin. Gastroenterol..

[92] García-Pagán J.C., Villanueva C., Vila M.C., Albillos A., Genescà J., Ruiz-Del-Arbol L., Rodriguez M., Calleja J.L., González A., Solà R. (2001). Isosorbide Mononitrate in the Prevention of First Variceal Bleed in Patients Who Cannot Receive β-blockers. Gastroenterology.

[93] Ge P.S., Runyon B.A. (2014). The changing role of beta-blocker therapy in patients with cirrhosis. J. Hepatol..

[94] D’Amico G., Pagliaro L., Bosch J. (1995). The treatment of portal hypertension: A meta-analytic review. Hepatology.

[95] Tandon P., Abraldes J.G., Berzigotti A., Garcia-Pagan J.C., Bosch J. (2010). Renin–angiotensin–aldosterone inhibitors in the reduction of portal pressure: A systematic review and meta-analysis. J. Hepatol..

[96] Hennenberg M., Trebicka J., Sauerbruch T., Heller J. (2008). Mechanisms of extrahepatic vasodilation in portal hypertension. Gut.

[97] Lugo-Baruqui A., Muñoz-Valle J.F., Arévalo-Gallegos S., Armendáriz-Borunda J. (2010). Role of angiotensin II in liver fibrosis-induced portal hypertension and therapeutic implications. Hepatol. Res..

[98] Silva A.C.S.E., Miranda A.S., Rocha N.P., Teixeira A.L. (2017). Renin angiotensin system in liver diseases: Friend or foe?. World J. Gastroenterol..

[99] Grace J.A., Klein S., Herath C.B., Granzow M., Schierwagen R., Masing N., Walther T., Sauerbruch T., Burrell L.M., Angus J.A. (2013). Activation of the Mas Receptor by Angiotensin-(1–7) in the Renin–Angiotensin System Mediates Mesenteric Vasodilatation in Cirrhosis. Gastroenterology.

[100] Gunarathne L.S., Angus P.W., Herath C.B. (2019). Blockade of Mas Receptor or Mas-Related G-Protein Coupled Receptor Type D Reduces Portal Pressure in Cirrhotic but Not in Non-cirrhotic Portal Hypertensive Rats. Front. Physiol..

[101] Herath C.B., Lubel J.S., Jia Z., Velkoska E., Casley D., Brown L., Tikellis C., Burrell L.M., Angus P.W. (2009). Portal pressure responses and angiotensin peptide production in rat liver are determined by relative activity of ACE and ACE2. Am. J. Physiol. Liver Physiol..

[102] Gunarathne L.S., Rajapaksha H., Shackel N., Angus P.W., Herath C.B. (2020). Cirrhotic portal hypertension: From pathophysiology to novel therapeutics. World J. Gastroenterol..

[103] Yoshiji H., Yoshii J., Ikenaka Y., Noguchi R., Tsujinoue H., Nakatani T., Imazu H., Yanase K., Kuriyama S., Fukui H. (2002). Inhibition of renin-angiotensin system attenuates liver enzyme-altered preneoplastic lesions and fibrosis development in rats. J. Hepatol..

[104] Wei H.S., Lu H.M., Li D.G., Zhan Y.T., Wang Z.R., Huang X., Cheng J.L., Xu Q.F. (2000). The regulatory role of AT1 receptor on activated HSCs in hepatic fibrogenesis: Effects of RAS inhibitors on hepatic fibrosis induced by CCl4. World J. Gastroenterol..

[105] Macgilchrist A.J., Howes L.G., Hawksby C., Reid J.L. (1991). Plasma noradrenaline in cirrhosis: A study of kinetics and temporal relationship to ascites formation. Eur. J. Clin. Investig..

[106] Ferlitsch A., Pleiner J., Mittermayer F., Schaller G., Homoncik M., Peck-Radosavljevic M., Wolzt M. (2005). Vasoconstrictor hyporeactivity can be reversed by antioxidants in patients with advanced alcoholic cirrhosis of the liver and ascites. Crit. Care Med..

[107] Angus P.W. (2006). Role of endothelin in systemic and portal resistance in cirrhosis. Gut.

[108] Miñano C., Garcia-Tsao G. (2010). Clinical Pharmacology of Portal Hypertension. Gastroenterol. Clin. North Am..

[109] Sieber C.C., Groszmann R.J. (1992). In vitro hyporeactivity to methoxamine in portal hypertensive rats: Reversal by nitric oxide blockade. Am. J. Physiol. Liver Physiol..

[110] Newby D.E., Jalan R., Masumori S., Hayes P.C., Boon N.A., Webb D.J. (1998). Peripheral vascular tone in patients with cirrhosis: Role of the renin–angiotensin and sympathetic nervous systems. Cardiovasc. Res..

[111] Hennenberg M., Trebicka J., Biecker E., Schepke M., Sauerbruch T., Heller J. (2007). Vascular dysfunction in human and rat cirrhosis: Role of receptor-desensitizing and calcium-sensitizing proteins. Hepatology.

[112] Neef M., Biecker E., Heller J., Schepke M., Nischalke H.D., Wolff M., Spengler U., Reichen J., Sauerbruch T. (2003). Portal hypertension is associated with increased mRNA levels of vasopressor G-protein-coupled receptors in human hepatic arteries. Eur. J. Clin. Investig..

[113] Hennenberg M., Biecker E., Trebicka J., Jochem K., Zhou Q., Schmidt M., Jakobs K.H., Sauerbruch T., Heller J. (2006). Defective RhoA/Rho-kinase signaling contributes to vascular hypocontractility and vasodilation in cirrhotic rats. Gastroenterology.

[114] Hennenberg M., Trebicka J., Kohistani A.Z., Heller J., Sauerbruch T. (2009). Vascular hyporesponsiveness to angiotensin II in rats with CCl4-induced liver cirrhosis. Eur. J. Clin. Investig..

[115] Vilas-Boas W.W., Ribeiro-Oliveira A., Pereira R.M., da Cunha Ribeiro R., Almeida J., Nadu A.P., e Silva A.C.S., dos Santos R.A.S. (2009). Relationship between angiotensin-(1–7) and angiotensin II correlates with hemodynamic changes in human liver cirrhosis. World J. Gastroenterol..

[116] Quilley J., Fulton D., McGiff J.C. (1997). Hyperpolarizing Factors. Biochem. Pharmacol..

[117] Sacerdoti D., Gatta A., McGiff J.C. (2003). Role of cytochrome P450-dependent arachidonic acid metabolites in liver physiology and pathophysiology. Prostaglandins Other Lipid Mediat..

[118] Tox U., Steffen H.M. (2006). Impact of inhibitors of the Renin-Angiotensin-aldosterone system on liver fibrosis and portal hypertension. Curr. Med. Chem..

[119] Eriksson L.S., Kågedal B., Wahren J. (1984). Effects of captopril on hepatic venous pressure and blood flow in patients with liver cirrhosis. Am. J. Med..

[120] Baik S.K., Park D.H., Kim M.Y., Choi Y.J., Kim H.S., Lee D.K., Kwon S.O., Kim Y.J., Park J.W., Chang S.J. (2003). Captopril reduces portal pressure effectively in portal hypertensive patients with low portal venous velocity. J. Gastroenterol..

[121] Chiang H.T., Cheng J.S., Lin M., tseng W.S., Chang J.M., Lai K.H. (1995). Haemodynamic effects of enalaprilat on portal hypertension in patients with HBsAg-positive cirrhosis. J. Gastroenterol. Hepatol..

[122] Svoboda P., Ochmann J., Kantorová I. (1992). Effect of enalapril treatment and sclerotherapy of esophageal varices on hepatic hemodynamics in portal hypertension. Hepatogastroenterology.

[123] Gracia-Sancho J., Laviña B., Rodríguez-Vilarrupla A., García-Calderó H., Bosch J., García-Pagán J.C. (2007). Enhanced vasoconstrictor prostanoid production by sinusoidal endothelial cells increases portal perfusion pressure in cirrhotic rat livers. J. Hepatol..

[124] Schepke M., Wiest R., Flacke S., Heller J., Stoffel-Wagner B., Herold T., Ghauri M., Sauerbruch T. (2008). Irbesartan Plus Low-Dose Propranolol Versus Low-Dose Propranolol Alone in Cirrhosis: A Placebo-Controlled, Double-Blind Study. Am. J. Gastroenterol..

[125] Schepke M., Werner E., Biecker E., Schiedermaier P., Heller J., Neef M., Caselmann W.H., Sauerbruch T., Stoffel-Wagner B., Hofer U. (2001). Hemodynamic effects of the angiotensin II receptor antagonist irbesartan in patients with cirrhosis and portal hypertension. Gastroenterology.

[126] Klein S., Herath C.B., Schierwagen R., Grace J., Haltenhof T., Uschner F.E., Strassburg C.P., Sauerbruch T., Walther T., Angus P.W. (2015). Hemodynamic Effects of the Non-Peptidic Angiotensin-(1–7) Agonist AVE0991 in Liver Cirrhosis. PLoS ONE.

[127] Sansoè G., Aragno M., Mastrocola R., Restivo F., Mengozzi G., Smedile A., Rosina F., Danni O., Parola M., Rizzetto M. (2005). Neutral endopeptidase (EC 3.4.24.11) in cirrhotic liver: A new target to treat portal hypertension?. J. Hepatol..

